# Para Phimosis Leading to Glans Gangrene - A Devastating Preventable Complication

**DOI:** 10.4274/balkanmedj.2016.0677

**Published:** 2017-03-28

**Authors:** Ashok Kumar Sokhal, Durgesh Kumar Saini, Satyanarayan Sankhwar

**Affiliations:** 1 Department of Urology, King George’s Medical College, Uttar Pradesh, India

Paraphimosis as an alarming and preventable clinical entity leading to glans gangrene - a lesson to learn.

After ethics committee approval and informed consent, we report a 34-year-old male patient presented with a history of storage and voiding lower urinary tract symptoms over 1 year. The patient developed acute urinary retention 7 days previously in which per-urethral catheterization was attempted at the primary health centre. Emergency urinary diversion was done by supra-pubic catheter. The patient presented at the urology clinic with high-grade fever with chills and blackening of the glans with penile swelling and inability to reduce the preputial skin to its normal position after retraction that occurred during a per-urethral catheterization attempt.

On physical examination, pulse rate of 104/minute, and blood pressure of 110/64 mm Hg were recorded. On local examination, dry gangrene of the glans with penile shaft oedema with paraphimotic ring with sloughing of penile skin was present (Figures 1, 2). Blood and serum chemistry demonstrated anaemia (haemoglobin 8.6 GM%), leucocytosis (total leukocyte count 18000/mm^3^) and deranged renal function (serum creatinine 2.1 mg/dL), suggestive of systemic inflammatory response syndrome. Fasting blood sugar was 94 mg/dL. Screening for hepatitis B, hepatitis C and human immunodeficiency virus was negative. Urine culture was positive for Escherichia coli, having a colony count >105/ high power field, which was sensitive to piperacillin and tazobactam, levofloxacin, amikacin, meropenem, and colistin.

According to the clinical scenario of the sepsis and urine culture report, the patient was managed by a combination of intravenous piperacillin and tazobactam 4.5 gram (Alkem Laboratories Limited, India) 8-hourly, and levofloxacin 500 mg (Cipla Laboratories Limited, India) once a day. After 48 hours there was a clear delineation of the gangrene area of glans examined by the senior urologist, for which the patient underwent necrotic tissue debridement and glansectomy. Post-operative period was uneventful. Culture and sensitivity report of debrided tissue was sterile, probably due to antibiotics institution.

Paraphimosis is a devastating condition occurring when the foreskin is retracted over the glans and cannot be replaced. The constriction ring of preputial skin forms a tourniquet, leading to constriction of the distal penis and venous engorgement resulting in skin oedema and distal gangrene. Paraphimosis is managed by compressive reduction, multiple needle punctures or by dorsal slit/circumcision (1). Raman et al. (2) reported a similar case of coital paraphimosis leading to penile glans gangrene, which was managed by excision of the necrotic area.

In conclusion, preputial skin must be pulled into place to prevent paraphimosis development whenever encountered retracted. Paraphimosis should be managed immediately by either external compression, manual preputial repositioning, dorsal slit or circumcision.

## Figures and Tables

**Figure 1:**
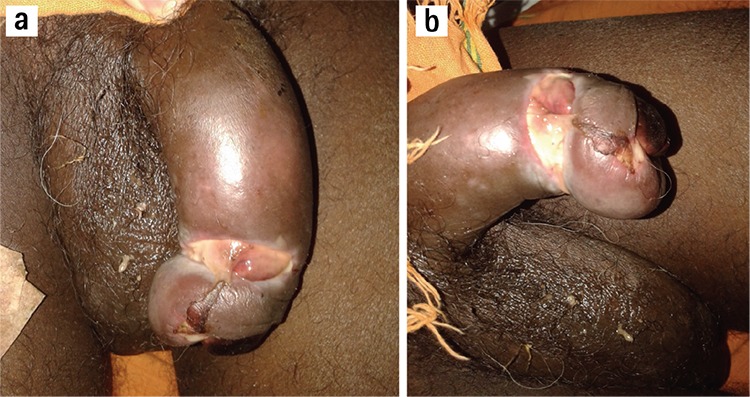
Black discoloration of glans penis with penile oedema (a). Dry gangrene of glans penis secondary to paraphimosis (b).

**Figure 2:**
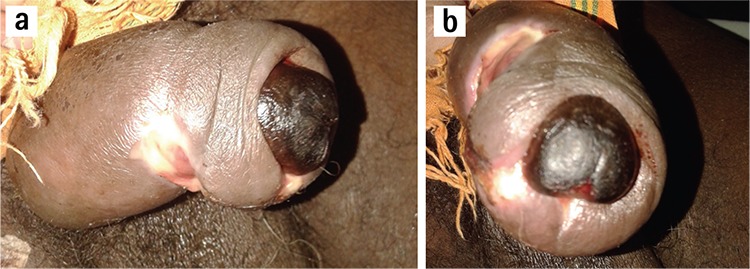
Demonstrating penile glans gangrene with paraphimotic constriction ring with purulent urethral discharge (a). Showing paraphimotic constriction ring with sloughed necrotic penile shaft skin (b).
